# Application of Stochastic Automata Networks for Creation of Continuous Time Markov Chain Models of Voltage Gating of Gap Junction Channels

**DOI:** 10.1155/2015/936295

**Published:** 2015-02-01

**Authors:** Mindaugas Snipas, Henrikas Pranevicius, Mindaugas Pranevicius, Osvaldas Pranevicius, Nerijus Paulauskas, Feliksas F. Bukauskas

**Affiliations:** ^1^Department of Mathematical Modelling, Kaunas University of Technology, Studentų Street 50, 51368 Kaunas, Lithuania; ^2^Laboratory of Systems Control and Automation, Lithuanian Energy Institute, Breslaujos Street 3, 44403 Kaunas, Lithuania; ^3^Department of Applied Informatics, Vytautas Magnus University, Vileikos Street 8-409, 44404 Kaunas, Lithuania; ^4^Department of Business Informatics Research in Systems, Kaunas University of Technology, Studentų Street 56, 5142 Kaunas, Lithuania; ^5^Department of Anesthesiology, Albert Einstein College of Medicine, 1300 Morris Park Avenue, Bronx, NY 10461, USA; ^6^Department of Anesthesiology, New York Hospital Queens, 56-45 Main Street, Flushing, NY 11355, USA; ^7^Institute of Cardiology, Lithuanian University of Health Sciences, Sukileliu Street 17, 50009 Kaunas, Lithuania; ^8^Department of Neuroscience, Albert Einstein College of Medicine, 1300 Morris Park Avenue, Bronx, NY 10461, USA

## Abstract

The primary goal of this work was to study advantages of numerical methods used for the creation of continuous time Markov chain models (CTMC) of voltage gating of gap junction (GJ) channels composed of connexin protein. This task was accomplished by describing gating of GJs using the formalism of the stochastic automata networks (SANs), which allowed for very efficient building and storing of infinitesimal generator of the CTMC that allowed to produce matrices of the models containing a distinct block structure. All of that allowed us to develop efficient numerical methods for a steady-state solution of CTMC models. This allowed us to accelerate CPU time, which is necessary to solve CTMC models, ∼20 times.

## 1. Introduction

Gap-junctional communication plays an important role in many processes, such as impulse propagation in the heart, communication between neurons and glia, organ formation during early development, regulation of cell proliferation, and metabolic exchange between cells of various tissues, including the lens that lack blood circulation. Gap junction (GJ) channels are formed of connexin (Cx) proteins, which belong to a family of integral membrane proteins exhibiting a tissue specific expression pattern. GJs provide a direct pathway for electrical and metabolic signalling between the cells [1]. In humans, twenty-one isoforms of Cxs form GJ channels [2]. Each GJ channel is composed of two hemichannels (HCs), both oligomerized of six Cxs. Cxs have four alpha helical transmembrane domains (M1 to M4), intracellular N- and C-termini (NT and CT), two extracellular loops (E1 and E2), and a cytoplasmic loop (CL) [3]. Docking of two HCs from neighbouring cells leads to formation of the GJ channel composed of 12 Cxs.

However, despite such complexity, all GJ channels share the same common property—sensitivity to transjunctional voltage (*V*_*j*_), called voltage gating. Junctional conductance (*g*_*j*_) measured under steady-state conditions decays symmetrically in response to *V*_*j*_ of either polarity, which have been explained by the presence of a *V*_*j*_-sensitive gate in each opposed HC [4]. Such property, being inherently quantitative, is amenable to the investigation by computational methods.

Earlier, we developed stochastic 4- and 16-state models of voltage gating, containing 2 and 4 gates in series in each GJ channel. In order to demonstrate that the proposed *V*_*j*_-gating model is adequate, it is necessary to compare its output to experimental results. For example, the proposed stochastic 4- and 16-state models of *V*_*j*_-gating contained a sizable number (>10) of parameters and for their estimation global optimization (GO) algorithms were successfully used [5, 6]. The simulation of *V*_*j*_-gating was performed with different sets of parameters. However, for the estimation of a global minimum, it typically requires running thousands of iterations, each lasting for up to 10 seconds, and consequently GO takes tens of hours or days. Thus, the reduction of the computation time that is needed for GO of experimental data is a critical task.

In previous work a discrete time Markov chain (DTMC) model was used [7]. This model described the GJ channel containing 12 gates. In such model, differently from the 4- and 16-state models, it was assumed that each Cx of the GJ channel contains the gate. Since all 12 gates operate at the same time, construction of the transition matrix is not a trivial task. Therefore, transition matrix **P** is dense and when direct methods, that is, Gaussian elimination, are applied, the run-time complexity of steady-state probabilities is in the neighbourhood of *O*(*n* ^3^). Numerical experiments showed that the use of DTMC model, as opposed to simulation, reduced CPU time ~18- and ~7-fold for 4- and 16-state models, respectively.

When using Markov chain models one needs to build the probability transition matrix and to estimate steady-state probabilities at different *V*_*j*_s at ~1000 different time moments during a single iteration. Typically, GO of experimental data require to use 500–5000 iterations to find a global minimum. Altogether, this will require to perform ~2 500 000 simulations using Markov model. Thus, it is evident that modeling requires fast construction of the matrix of transition probabilities (transition rates) and fast solution of the steady-state probabilities because the amount of central processing unit (CPU) time is high even at relatively small number of states. In our prior studies [8], we already used continuous time Markov chain (CTMC) model of GJs gating. A transformation of the transition probabilities into transition rates is necessary to generate CTMC model with the same steady-state probabilities, but infinitesimal generator matrix of CTMC model is sparse. For example, infinitesimal generator of GJ model presented in [8] was a tridiagonal matrix. Therefore, generation of matrix and modeling of GJs under steady-states conditions using the CTMC model require smaller amount of CPU time compared to using the DTMC model.

In the present study, we used CTMC for the modeling of GJs containing two voltage-sensitive gates, each of which is composed of six subgates attributed to each Cx; in stochastic 4- and 16-state gating models each gate is regarded as one unit. We also used a stochastic automata network (SAN) formalism for the Markov model specification. SAN formalism allowed accelerating generation of the transition-rate matrices.

In previous study [8] we used piece linear aggregate (PLA) formalism for CTMC model creation. PLA formalism allows building and storing infinitesimal generator of Markov chain model automatically, but matrix structure cannot be easily deduced, especially in more complex models. On the other hand, SAN formalism is a method that is based on using tensor algebra matrix operations. Consequentially, infinitesimal generators have the distinct structure allowing for very efficient application of the numerical methods. Here, we present SAN description of CTMC models of three different GJ models and analyze the efficiency of numerical solution. Since CPU time depends on software and its implementation, we focused on more universal evaluation of a complexity of algorithms. It is based on the exact number of mathematical operations, which is necessary to perform steady-state probabilities calculation.

Our studies showed that if a proper numerical method is applied, then a steady-state solution of the proposed CTMC model of GJs requires 10–20-fold less CPU time compared to DTMC models. We suggest that the use of iterative methods might be especially beneficial in estimation of gating parameters, since it requires repetitive simulations with different sets of parameters. We showed that using a previous solution for evaluating continuous one, when *V*_*j*_ changes are small (<1 mV), allows reducing the number of iterations for at least 30 percent.

## 2. Methods

### 2.1. Markov Chain Modeling

We assume the stationary analysis of a homogenous irreducible Markov chain with a finite number of system states, denoted by *n*. Markov chain modeling consists of two stages: (1) construction of transition-rates matrix called an infinitesimal generator and (2) calculation of steady-state probabilities.

The first stage is a model specification. For a GJ gating model this could mean defining the states of a single gate and possible transitions among them, the number of gates in the GJ, and so forth. Basically, it results in formation of a transition matrix **P**, if one assumes a DTMC, or infinitesimal generator matrix **Q**, if one assumes CTMC. In this paper we consider mainly the CTMC models.

Formation of **P** and **Q** can be performed manually if the size of state space (*n*) is relatively small. For larger models the special software can be used, for example, methods based on events language [9], Petri nets [10], and stochastic automata networks [11].

One of the main problems in Markov chain modeling of real systems is a rapid growth of the number of states. The number of states of the Markov chain grows exponentially, when the number of system components grows linearly. Therefore the use of efficient model creation tools and numerical methods is crucial [12].

### 2.2. Calculation of Steady-State Probabilities

The most difficult and time consuming part of Markov chain modeling is calculation of steady-state probabilities.

Computation of steady-state probabilities of DTMC, which are stored as a row-vector ***π***, is the solution of a system of linear equations:

(1) $$\begin{array}{c} \pi \cdot P=\pi . \end{array}$$

Similarly, computation of steady-state probabilities of DTMC, which are stored as a row-vector ***π***, is the solution of a system of linear equations:

(2) $$\begin{array}{c} \pi \cdot Q=0, \end{array}$$

where 0 denotes a zero row-vector of size *n*.

Equations (1)-(2) can also be interpreted as computations of left side eigenvector corresponding to eigenvalue 1 (in case of DTMC) or eigenvalue 0 (in case of CTMC). Since **P** and **Q** are singular, an additional condition ∑_*i*=1_^*n*^ ***π***_*i*_ = 1 is used in all cases.

There are three big classes of algorithms allowing evaluation of steady state probabilities: direct methods, iteration methods, and projection methods. More about numerical methods for solution of general linear systems can be found in [13]; more about application of numerical methods specifically for Markov chains is in [14].

### 2.3. Stochastic Automata Networks

SAN formalism [15] is one of the most efficient methods used to solve state-space explosion problem, which is very detrimental in Markov chain modeling. SAN allows very efficient construction and storage of infinitesimal generator **Q** by using tensor (Kronecker) algebra operations.

Though SAN formalism originally was developed for the modeling of computer networks and communication systems [16, 17], there are multiple examples of SAN use in biology. For example, DeRemigio et al. [18] and Hao et al. [19] used SANs formalism to model calcium channels; Wolf [20] used SANs to describe kinetics of biochemical reactions.

The main idea of SAN formalism is based on the division of a system into smaller subsystems, which can interact among themselves. Those subsystems are described by different stochastic automata. A single automaton is represented by a Markov chain, that is, by the set of subsystem states and possible transitions among them. If two (or more) automata somehow interact among themselves, then transition in one automaton may depend on the state of another one.

The state of the whole system, so called global state, is a compositional state of all automata. An infinitesimal generator matrix of the whole system, so called global generator matrix, can be expressed by infinitesimal generators of individual automata and Kronecker algebra operations. We recall basic definitions below.

Kronecker product **A** ⊗ **B** of two matrices **A** ∈ *R* ^*m*×*n*^ and **B** ∈ *R* ^*p*×*q*^ is given by

(3) $$\begin{array}{c} A⊗B=\left(\begin{array}{ccc} a_{11}B & \cdots & a_{1n}B\\ \vdots & \ddots & \vdots \\ a_{m1}B & \cdots & a_{mn}B \end{array}\right)\in R^{mp\times nq}. \end{array}$$

Kronecker sum **A** ⊕ **B** of two squared matrices **A** ∈ *R* ^*m*×*m*^ and **B** ∈ *R* ^*n*×*n*^ is given by

(4) $$\begin{array}{c} A⊕B=A⊗I_{n}+I_{m}⊗B\in R^{mn\times mn}, \end{array}$$

where **I**_*n*_, **I**_*m*_ are identity matrices of sizes *n* and *m*, respectively. The Kronecker sum of more than two square matrices is also well defined [21].

If the network consists of *k* independent stochastic automata *A* ^(*i*)^, each governed by infinitesimal generators **Q** ^(*i*)^, *i* = 1 ⋯ *k*, then the global infinitesimal generator **Q** can be expressed as a tensor sum:

(5) $$\begin{array}{c} Q\;=\overset{k}{\underset{i=1}{⨁}}Q^{\left(i\right)}. \end{array}$$

Expression (5) is also called the SAN descriptor of the system. For a SAN of independent automata steady-state probability, the vector ***π*** of the whole system is given by

(6) $$\begin{array}{c} \pi =\;\overset{k}{\underset{i=1}{⨂}}\pi^{\left(i\right)}, \end{array}$$

where ***π*** ^(*i*)^ is a steady-state probability vector of an individual automaton *A* ^(*i*)^.

If we are to consider a network, describing two independent gates that operate between open and closed states with transition rates *λ*_*oc*_ and *λ*_*co*_, then automata *A* ^(1)^ and *A* ^(2)^ model each hemichannel/gate, and each of them can be described using the infinitesimal generator:

(7) $$\begin{array}{c} Q^{\left(1\right)}=Q^{\left(2\right)}=\left(\begin{array}{cc} -\lambda_{oc} & \lambda_{oc}\\ \lambda_{co} & -\lambda_{co} \end{array}\right). \end{array}$$

Thus, the SAN descriptor of the network of two independent gates is given by

(8) Q=Q1⊕Q2=Q1⊗I2+I2⊗Q2=−2λocλocλoc0λco−λco−λoc0λocλco0−λco−λocλoc0λcoλco−2λco.

If automata in SAN are not completely independent, the interaction among them can also be expressed by the use of Kronecker algebra operations. Plateau expressed two different ways [15] to describe the interaction among automata.

*(1) Functional Transition Rates*. A transition rate in a single automaton may depend on the state of the other automata, that is, on the global system state. Transition rates, which are independent on the global system state, are called constant transition rates.

If we are to consider a network composed of 2 automata and suppose that the transition rates of each connexin depend on the number of connexins/subgates in the open state (denoted by *n*_*o*_), then transition rates are functions: *λ*_*oc*_ = *λ*_*oc*_(*n*_*o*_) and *λ*_*co*_ = *λ*_*co*_(*n*_*o*_). Consequently, the SAN descriptor of the system is as follows:

(9) Q=Q1⊕gQ2=−2λoc2λoc2λoc20λco1−λco1−λoc10λoc1λco10−λco1−λoc1λoc10λco0λco0−2λco0,

where ⊕_*g*_ denotes the generalized Kronecker product, which deals with functional transition rates [22].

*(2) Synchronizing Events*. Transition in one automaton can cause transitions in other automata. Transition rates are called local, if they are not transition rates of synchronizing events. Synchronizing transitions may also be functional. In this paper, we do not use synchronizing events for the creation of gap junction models.

Plateau and Atif showed that the SAN descriptor of a network consisting of *k* automata and having *l* synchronizing events can be expressed as follows:

(10) $$\begin{array}{c} Q=\overset{k+2l}{\underset{j=1}{\sum }}\overset{k}{\underset{\begin{array}{c} i=1\\ g \end{array}}{⨂}}Q_{j}^{\left(i\right)}. \end{array}$$

The use of SAN formalism basically solves matrix construction problems, since even large matrices can be built and stored (assuming there is enough storage space in operative memory) in a very short time.

The main problem that arises when dealing with SANs of interacting automata is that the steady-state solution cannot be expressed as a simple product form (6). In this case, steady-state probabilities can be found either from solving (2) after **Q** is built from the SAN descriptor or directly from the descriptor. That is, building and storing of **Q** is not necessary, if special numerical methods are applied. It is possible not to build **Q**, since vector SAN descriptor product ***π***(∑_*j*=1_^*T*^⊗_*i*=1_^*N*^ **Q**_*j*_^(*i*)^) can be implemented efficiently, for example, by using shuffle algorithm. These problems are considered in detail in [23].

## 3. Results and Discussion

In this section, we present CTMC models of GJs, created by using SAN formalism. The structure of infinitesimal generators and efficient application of numerical methods for steady-state solutions are considered in detail.

### 3.1. CTMC Model of the GJ Channel Containing the 12 Two-State Subgates

**Figure 1:**
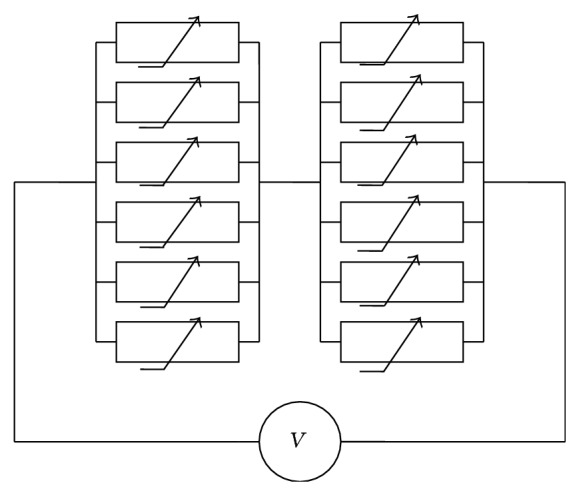
Electrical scheme of the GJ channel composed of two hemichannels each formed of 6 connexins. Transjunctional voltage (*V*_*j*_) controls both hemichannels and all Cxs can operate between open and closed (and deep closed) states.

Gap junctions form clusters (junctional plaques) of individual channels arranged in parallel in the junctional membrane of two adjacent cells. The GJ channel is composed of 2 hemichannels (left and right) arranged in series. Each hemichannel is composed/oligomerized from six Cxs forming a hexamer with the pore inside. We envision that each hemichannel forms the gate, which is composed of six subgates arranged in parallel; that is, to each connexin the subgate is attributed and the GJ channel ultimately contains two gates composed of 12 subgates (see Figure 1).

**Figure 2:**
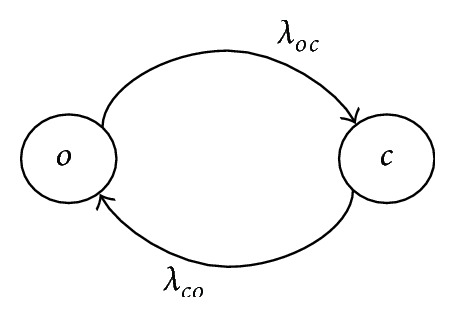
Two-state transition graph of a subgate.

In this section, we will consider a model, in which each subgate operates between open (*o*) and closed (*c*) states (see Figure 2), with transition rates *λ*_*oc*_ (from *o* to *c*) and *λ*_*co*_ (from *c* to *o*).

One of the most important steps of SAN model creation is to decide which part of a system to model by an individual automaton. In this case it is possible to describe each subgate as an individual automaton with two states. This would result in SAN of 12 automata with 2^12^ = 4096 states.

However, it is unnecessary to track each subgate individually; since all subgates are identical then *V*_*j*_-gating depends on the number of open and closed gates in each hemichannel. Thus, much more convenient way is to describe the whole hemichannel as an individual automaton, whose states denote the number of closed (or open) subgates in hemichannel.

Thus we model the GJ channel by two automata—an automaton *A*_2_^(*l*)^, which describes the left hemichannel, and *A*_2_^(*r*)^, which describes the right hemichannel (number 2 in the subscript denotes the fact, that each subgate has two possible states).

We assume that both automata have 7 possible states, which denote the number of closed subgates in each hemichannel (i.e., it can be denoted by 0,1,…, 6).

Thus, automaton *A*_2_^(*l*)^ can leave a state $n_{l}\;\;\left(n_{l}=\bar{1,6}\right)$ and enter a state *n*_*l*_ − 1 with transition rate *n*_*l*_ · *λ*_*co*_. Similarly, it can leave state $n_{l}\;\;\left(n_{l}=\bar{0,5}\right)$ and go to the state *n*_*l*_ + 1 with transition rate (6 − *n*_*l*_) · *λ*_*oc*_.

Thus an infinitesimal generator **Q**_2_^(*l*)^ of automaton *A*_2_^(*l*)^ is as follows:

(11) $$\begin{array}{c} Q_{\left(2\right)}^{\left(l\right)}=\left(\begin{array}{ccccccc} {}* & 6\lambda_{oc} & 0 & 0 & 0 & 0 & 0\\ \lambda_{co} & * & 5\lambda_{oc} & 0 & 0 & 0 & 0\\ 0 & 2\lambda_{co} & * & 4\lambda_{oc} & 0 & 0 & 0\\ 0 & 0 & 3\lambda_{co} & * & 3\lambda_{oc} & 0 & 0\\ 0 & 0 & 0 & 4\lambda_{co} & * & 2\lambda_{oc} & 0\\ 0 & 0 & 0 & 0 & 5\lambda_{co} & * & \lambda_{oc}\\ 0 & 0 & 0 & 0 & 0 & 6\lambda_{co} & * \end{array}\right), \end{array}$$

where diagonal entries (denoted by ∗) are equal to the negated sum of the nondiagonal entries in that row.

It is crucial to emphasize that transition rates of the matrix **Q**_(2)_^(*l*)^ in (11) depend on the voltage across the left and right hemichannels, which accordingly depends on the number of closed (open) gates:

(12) λoc=λocVleftnl,Vrightnr,λco=λcoVleftnl,Vrightnr.

Since both hemichannels are identical, an infinitesimal generator of the right hemichannel is the same; that is, **Q**_(2)_^(*l*)^ = **Q**_(2)_^(*r*)^. In this case, global infinitesimal generator of the GJ, which we denote by **Q**_2_^(12)^, may be written as

(13) $$\begin{array}{c} Q_{\left(2\right)}^{\left(12\right)}=Q_{2}^{\left(l\right)}\underset{g}{⊕}Q_{2}^{\left(r\right)}=Q_{2}^{\left(l\right)}\underset{g}{⊗}I_{7}+I_{7}\underset{g}{⊗}Q_{2}^{\left(r\right)}. \end{array}$$

Since both **Q**_2_^(*l*)^ and **Q**_2_^(*r*)^ are tridiagonal matrices, it follows from (13) that **Q**_2_^(12)^ is a block tridiagonal matrix. It consists of 49 square blocks, each of size 7 and can be written as follows:

(14) $$\begin{array}{c} Q_{\left(2\right)}^{\left(12\right)}=\left(\begin{array}{ccccccc} Q_{11} & Q_{12} & 0 & 0 & 0 & 0 & 0\\ Q_{21} & Q_{22} & Q_{23} & 0 & 0 & 0 & 0\\ 0 & Q_{32} & Q_{33} & Q_{34} & 0 & 0 & 0\\ 0 & 0 & Q_{43} & Q_{44} & Q_{45} & 0 & 0\\ 0 & 0 & 0 & Q_{54} & Q_{55} & Q_{56} & 0\\ 0 & 0 & 0 & 0 & Q_{65} & Q_{66} & Q_{76}\\ 0 & 0 & 0 & 0 & 0 & Q_{76} & Q_{77} \end{array}\right). \end{array}$$

Here 0 denote square zero matrix blocks of size 7.

Since **Q**_(2)_^(*l*)^ and **Q**_(2)_^(*r*)^ are tridiagonal matrices, then from (14) it follows that diagonal blocks **Q**_*ii*_ are the following tridiagonal matrices:

(15) $$\begin{array}{c} Q_{ii}=-\left(\left(i-1\right)\lambda_{co}+\left(7-i\right)\lambda_{oc}\right)I_{7}+Q_{2}^{\left(r\right)},\;i=\bar{1,7}. \end{array}$$

Here *λ*_*co*_ = *λ*_*co*_(*V*_left_(7 − *i*), *V*_right_(*j*)) and *j* is the row in a block **Q**_*ii*_, in which transition rate *λ*_*co*_ appears. Similarly, *λ*_*oc*_ = *λ*_*oc*_(*V*_left_(7 − *i*), *V*_right_(*j*)) and *j* is the row in a block **Q**_*ii*_, in which *λ*_*oc*_ appears.

Similarly, upper subdiagonal blocks in **Q**_*i*+1,*i*_ may be written as follows:

(16) $$\begin{array}{c} Q_{i,i+1}=\left(7-i\right)\lambda_{oc}I_{7},\;i=\bar{1,6}, \end{array}$$

where *λ*_*oc*_ = *λ*_*oc*_(*V*_left_(7 − *i*), *V*_right_(*j*)) and *j* is the* row* in a block **Q**_*i*,*i*+1_, in which *λ*_*oc*_ appears.

And finally, lower subdiagonal blocks in (14) are as follows:

(17) $$\begin{array}{c} Q_{i+1,i}=\left(i-1\right)\lambda_{co}I_{7},\;i=\bar{2,7}, \end{array}$$

where *λ*_*co*_ = *λ*_*co*_(*V*_left_(*i* + 1), *V*_right_(*j*)) and *j* is the row in a block **Q**_*i*,*i*+1_, in which *λ*_*co*_ appears.

### 3.2. Evaluation of Functional Transition Rates

As we mentioned in a previous chapter, all transition rates in GJ voltage gating model are functional. Therefore, each transition changes the number of closed (open) gates, which changes the conductance and voltage across the channel accordingly, thus changing the values of transition rates. These changes depend on gating parameters of subgates; in homotypic GJ channels they are identical for all 12 subgates.

Even though these formulas were published earlier [6], we present them here, since they demonstrate the complexity of functional transition rates using SAN modeling of GJ.

In the DTMC model, probabilities of two-state gate transitions can be described as follows:

(18) $$\begin{array}{c} p_{oc}\left(A,P,V_{\text{left}\left(\text{right}\right)},V_{0}\right)=\frac{K\cdot k\left(A,P,V_{\text{left}\left(\text{right}\right)},V_{0}\right)}{1+k\left(A,P,V_{\text{left}\left(\text{right}\right)},V_{0}\right)},\\ p_{co}\left(A,P,V_{\text{left}\left(\text{righ}\right)t},V_{0}\right)=\frac{K}{1+k\left(A,P,V_{\text{left}\left(\text{right}\right)},V_{0}\right)}. \end{array}$$

In (18), *k* is

(19) $$\begin{array}{c} k\left(P,V_{\text{left}\left(\text{right}\right)},V_{0}\right)=e^{A\cdot \left(P\cdot V_{\text{left}\left(\text{right}\right)}-V_{0}\right)}, \end{array}$$

where *P* is a gating polarity (+1 or −1); *A* (1/mV) is a coefficient characterizing gating sensitivity to voltage; *K* is a constant used to change kinetics of *c*↔*o* transitions (*K* can accelerate or decelerate *c*↔*o* transitions but does not affect conditions of the steady-state); *V*_*o*_ (mV) is a voltage across the hemichannel/connexin at which probabilities for *o* and *c* states are equal; *V*_left/right_ is variable voltage across the left or right subgate (mV).

DTMC probabilities can be transformed into transition rates of CTMC by the following equation:

(20) $$\begin{array}{c} \lambda_{oc}=\frac{p_{oc}}{\tau },\;\;\lambda_{co}=\frac{p_{co}}{\tau }, \end{array}$$

where *τ* is a short period of time, in which the probability to observe multiple transitions is negligible; that is, for *i* ≠ *j*, *p*_*ij*_(*τ*) → 0 if *τ* → 0.

Each subgate, depending on a voltage across it (*V*_left/right_), can gate/operate by changing stepwise between the open state with conductance *g*_*o*_ and the closed state with conductance *g*_*c*_. It was assumed that *g*_*o*_ and *g*_*c*_ values exhibit rectification, that is, depend on *V*_left/right_ exponentially:

(21) $$\begin{array}{c} g_{o}\left(V_{\text{left}\left(\text{right}\right)},P\right)=2\cdot \exp\left\{\frac{P\cdot V_{\text{left}\left(\text{right}\right)}}{R_{o}}\right\},\\ g_{c}\left(V_{\text{left}\left(\text{right}\right)},P\right)=0.25\cdot \exp\left\{\frac{P\cdot V_{\text{left}\left(\text{right}\right)}}{R_{c}}\right\}, \end{array}$$

where *V*_left/right_ is a voltage across the left or right hemichannel, while *R*_*o*_ and *R*_*c*_ are rectification constants.

The conductance of the left hemichannel, when *n*_*l*_ Cxs are closed, can be described as follows:

(22) $$\begin{array}{c} g_{\text{left}}\left(n_{l}\right)=n_{l}g_{c}\left(V_{\text{left}}\left(n_{l}\right),P\right)+\left(6-n_{l}\right)g_{o}\left(V_{\text{left}}\left(n_{l}\right),P\right). \end{array}$$

Similarly, the conductance of the right hemichannel, when *n*_*r*_ Cxs are closed, is

(23) $$\begin{array}{c} g_{\text{right}}\left(n\right)=n_{r}g_{c}\left(V_{\text{right}}\left(n\right),P\right)+\left(6-n_{r}\right)g_{o}\left(V_{\text{right}}\left(n\right),P\right). \end{array}$$

During gating, conductance of subgates ranges between *g*_*o*_(*V*_left(right)_, *P*) and *g*_*c*_(*V*_left(right)_, *P*), and the total conductance of the GJ channel can be found using steady-state probabilities of Markov-chain model:

(24) $$\begin{array}{c} g=\sum_{i}\pi_{i}\frac{g_{\text{left}}\left(V_{\text{left}}\left(n_{l}\right)\right)\cdot g_{\text{right}}\left(V_{\text{right}}\left(n_{r}\right)\right)}{g_{\text{left}}\left(V_{\text{left}}\left(n_{l}\right)\right)+g_{\text{right}}\left(V_{\text{right}}\left(n_{r}\right)\right)}, \end{array}$$

where *π*_*i*_ is a steady-state probability for *n*_*l*_ Cxs in the left hemichannel and *n*_*r*_ Cxs in the left hemichannel to be closed.

Conductance of the GJ channel depends on the voltage; that is, the circuit is nonlinear. In order to calculate voltage across each Cx, we used an iterative procedure [7]. We assumed that the value of voltage is settled, if a difference between voltage values, calculated at two consecutive iterations, is less than 0.1 percent. Calculation showed that at least 5 iterations were needed to achieve the aforesaid precision.

As one can see, the evaluation of functional transition rates of SAN model is not a trivial task in this case. SAN formalism allows estimating steady-state probabilities directly from SAN descriptor but not building and storing infinitesimal generator, for example, by using shuffle algorithm. However, there would be a necessity to evaluate functional transition rates during each iteration for evaluation of steady-state probabilities. Thus, it would require too much CPU time in case of GJ models. Moreover, the number of system states of GJ models presented in this paper is relatively small; therefore we use the different approach. We apply SAN formalism to specify the system behaviour and to create equivalent Markov chain model. The use of Kronecker algebra representation of global infinitesimal generator helps to get the insight of matrix structure and to apply numerical methods for evaluation of a steady-state solution.

### 3.3. Numerical Solution of Two-State CTMC Models of the GJ Channel Containing 12 Two-State Subgates

It follows from (16)-(17) that subdiagonal blocks in (14) are diagonal matrices. These properties allow using numerical methods for the calculation of steady-state probabilities and we consider three algorithms for steady-state solution in detail: banded Gaussian elimination, direct solution by recursion, and block Gauss-Seidel methods.

#### 3.3.1. Banded Gaussian Elimination for Steady-State Solution of the CTMC Model of GJ Channel Containing 12 Two-State Subgates

A square matrix **Q** = (*q*_*ij*_) is called banded if its entries are zero outside of the diagonally bordered band, which can be described by the following equation:

(25) $$\begin{array}{c} q_{ij}=0,\;\text{if}\;\;j<i-k_{1}\;\;\text{or}\;\;j>i+k_{2}. \end{array}$$

In (25), numbers *k*_1_ and *k*_2_ are called left and right half-bandwidths, respectively. The bandwidth of the matrix *m* is equal to (*k*_1_ + *k*_2_ + 1). For example, a matrix with *k*_1_ = *k*_2_ = 1 is tridiagonal matrix, that is, matrix with bandwidth 3.

*Complexity*. If a matrix has bandwidth *m*, a more efficient implementation of Gaussian elimination exists than the standard one, which has a complexity of *O*(*n* ^3^). The solution of the linear system with bandwidth *m* has an approximate complexity of *O*(*m* ^2^ *n*). To be exact, the complexity of banded Gaussian elimination is *n*(*m* + 1)^2^/4, while it is (4*n* ^3^ + 9*n* ^2^ − 13*n*)/6 for standard Gaussian elimination [24].

From (14) it follows that **Q** has bandwidth *m* = 15. Thus, it requires approximately 13 times less CPU time to apply banded Gaussian elimination to solve an equivalent DTMC model, which has a dense transition probability matrix.

#### 3.3.2. Recursive Method for Steady-State Solution of CTMC Model of GJ Channel Containing 12 Two-State Subgates

An algorithm similar to the Thomas algorithm for tridiagonal matrices can be used to calculate steady-state probabilities of the GJ model. We use the matrix form of solution technique as presented in [14]. Infinitesimal generator **Q** can be divided into four blocks as follows:

(26) $$\begin{array}{c} V^{\left(l\right)}={\left(\begin{array}{cccccc} Q_{11} & Q_{21} & 0 & 0 & 0 & 0 \end{array}\right)}^{T},\\ X=\left(0\right),\;\;Y=\left(\begin{array}{cccccc} 0 & 0 & 0 & 0 & Q_{76} & Q_{77} \end{array}\right),\\ W=\left(\begin{array}{cccccc} Q_{12} & 0 & 0 & 0 & 0 & 0\\ Q_{22} & Q_{23} & 0 & 0 & 0 & 0\\ Q_{32} & Q_{33} & Q_{34} & 0 & 0 & 0\\ 0 & Q_{43} & Q_{44} & Q_{45} & 0 & 0\\ 0 & 0 & Q_{54} & Q_{55} & Q_{56} & 0\\ 0 & 0 & 0 & Q_{65} & Q_{66} & Q_{67} \end{array}\right). \end{array}$$

Dividing vector ***π*** into segments, solution of

(27) $$\begin{array}{c} \left(\begin{array}{cc} \pi_{*} & \pi_{7} \end{array}\right)\left(\begin{array}{cc} V & W\\ X & Y \end{array}\right)=0 \end{array}$$

can be implemented by solving ***π***_7_ **Y** **W** ^−1^ **V** = 0 in two steps. At first, solving **W** **Z** = **V** for **Z** gives **W** ^−1^ **V**, while **Y** **Z** gives coefficient matrix for solving ***π***_7_. After that, the remaining part ***π***_∗_ of steady-state vector ***π*** can be found from ***π***_∗_ **W** = ***π***_7_ **Y**.

*Complexity.* The procedure for obtaining a recursive solution can be implemented very efficiently due to the structure of the infinitesimal generator **Q**. For example, computation of **Z** is basically a backward substitution, since **W** is lower triangular. Also, finding the inverse of subdiagonal blocks is a trivial task, since these blocks are diagonal matrices.

Since the size of state-space *n* is not large for this Markov model, an approximate complexity evaluation by using big *O* notation might be too general in this case. Therefore, we evaluated the amount of operations necessary to implement the recursive procedure in more detailed way. We distinguished 6 types of distinct operations (e.g., matrix summation, matrix multiplication, etc.) adapted to different types of operands, such as dense matrix and tridiagonal matrix. A conservative estimation of number of arithmetic operations is presented in Table 1. We assume that these operations are implemented in the most basic way (e.g., we use (*c*_*ij*_) = ∑_*i*=1_^*n*^ *a*_*ik*_ *b*_*kj*_ for matrix multiplication **C** = **A** **B**).

Detailed evaluation showed that recursive solution requires about 1.8 times less CPU time than banded Gaussian elimination and at least 23 times less than standard Gaussian elimination.

*Stability*. The main problem of the block recursive procedure is numerical stability, due to rounding errors [14]. However, these problems are mitigated by the fact that matrix entries are relatively small (in the range of 0.001–0.01).

We compared the solution provided by the recursive procedure with the solution calculated using a stable numerical method. Experimental data showed that steady-state probabilities obtained by the recursive procedure differ less than 10^−12^ from an exact solution for this particular GJ model.

#### 3.3.3. Block Gauss-Seidel Method for Steady-State Solution of CTMC Model of GJ Channels Containing 12 Two-State Subgates

The block Gauss-Seidel method is an iterative technique and thus eliminates stability concerns completely [14]. It can also be implemented very efficiently for block tridiagonal matrices.

If solution vector ***π*** is divided according to the block structure of **Q**, then at *k* + 1 outer iteration it is required to solve 7 inner iterations, which may be written as follows:

(28) $$\begin{array}{c} \pi_{1}^{\left(k+1\right)}=-\left(\pi_{2}^{\left(k\right)}Q_{21}\right)Q_{11}^{-1};\\ \pi_{i}^{\left(k+1\right)}=-\left(\pi_{i-1}^{\left(k+1\right)}Q_{i-1,1}+\pi_{i+1}^{\left(k\right)}Q_{i+1,1}\right)Q_{11}^{-1},\;i=\bar{2,6};\\ \pi_{7}^{\left(k+1\right)}=-\left(\pi_{6}^{\left(k+1\right)}Q_{67}\right)Q_{77}^{-1}. \end{array}$$

*Complexity*. In this case, diagonal blocks are tridiagonal matrices and the linear system solution has a complexity of *O*(*n*). The number of operations (including a proof for convergence) necessary to perform a single outer iteration is presented in Table 2.

The efficiency of the whole algorithm depends on convergence speed. That is, block Gauss-Seidel method would be more efficient than the recursive procedure if it required 4 or less iterations. Similarly, it would be more efficient than banded Gaussian elimination if the number of outer iterations was less than 7, and, finally, it would be more efficient than standard Gaussian elimination if block Gauss-Seidel required less than 96 outer iterations.

We evaluated the number of outer iterations necessary to find a steady-state solution of the GJ model containing 12 two-state gates at *V*_*j*_ = 40 mV. Each time an initial iteration vector was chosen as a standard vector with equal entries, that is, each entry equal to 1/*n*. We assumed that necessary precision *ε* is achieved if the following condition was satisfied:

(29) $$\begin{array}{c} \underset{i}{\max}\left|\pi_{i}^{\left(k+1\right)}-\pi_{i}^{\left(k\right)}\right|<\varepsilon . \end{array}$$

Inequality (29) means that all entries of consecutive iterations differ less than *ε* in absolute value. The results of a convergence are presented in Table 3.

Thus, for this particular GJ model the block Gauss-Seidel method is more efficient than standard Gaussian elimination at any precision level. However it is less efficient than the direct methods, for example, banded Gaussian algorithm or the recursive procedure presented in Section 3.3.2.

In addition, modification of the block Gauss-Seidel method outperformed other standard iterative algorithms (Jacobi, Gauss-Seidel) as well as projection methods (Arnoldi, GMRES, and BiCGSTAB). While the convergence of the fastest of projection methods, BiCGSTAB, was slightly better than that of block Gauss-Seidel, overall CPU time was longer (about 18 percent), since it required more time to perform a single iteration.

#### 3.3.4. Repetitive Model Solution

Moreover, since the block Gauss-Seidel method is an iterative technique, it can be implemented more efficiently for repetitive solutions, which are especially important in the search for optimal model parameters. Iterative methods have an advantage over direct methods, since the previous solution vector can be used as a starting point for the solution of model with different set of parameters.

A solution of the model at different *V*_*j*_ values can be described using Pseudocode 1.

A similar procedure can be applied not only at different *V*_*j*_s, but also with other gating parameters as well.

It is known [25] that the difference between steady-state solutions of two linear systems, **Q** · ***π*** = 0 and (**Q** + Δ**Q**) · (***π*** + Δ***π***) = 0, is constrained by the following relationship between norms of matrices and vectors:

(30) $$\begin{array}{c} \frac{\left\|\Delta \pi \right\|}{\left\|\pi \right\|}\leq \left\|Q^{\#}\right\|\cdot \left\|\Delta Q\right\|; \end{array}$$

**Q** ^#^ is group inverse of infinitesimal generator **Q**.

Thus, steady-state vectors ***π*** ^(*i*)^ are relatively close to each other if matrices **Q** and (**Q** + Δ**Q**) are close and ‖**Q** ^#^‖ is not large.

Simulation of *V*_*j*_-gating partially satisfies aforementioned conditions, since changes of entries of **Q** are in the range of 0.0001–0.001 due to the voltage change from 40 mV to 41 mV, while the norm ‖**Q** ^#^‖ is equal to 213.73 at 40 mV level.

In order to evaluate an effect of iterative methods for the repetitive model solution, more detailed experimental research was performed. We changed voltage across GJ channel from 0 to 100 mV by 0.1 mV intervals. Thus, 1000 different infinitesimal generators were built and the steady-state solution was calculated according to a scheme presented in Pseudocode 1. Firstly, we used standard iteration vector with equal entries each time (we will refer to it as “Method I”), while for the second round of calculation a previous steady-state solution was used each time, as presented in Pseudocode 1 (we will refer to it as “Method II”). We evaluated the number of outer iterations necessary to achieve 10^−6^ precision for both cases. The results are presented in Table 4.

A positive effect of “Method II” is obvious. The number of outer iterations decreases from about 30 to 60 percent depending on *V*_*j*_ level. Overall, it required about 38 percent less CPU time to perform calculations by using “Method II” instead of “Method I.” CPU time in this case was comparable to that of banded Gaussian elimination.

### 3.4. CTMC Model of the GJ Channel Containing 6 Three-State Gates

**Figure 3:**
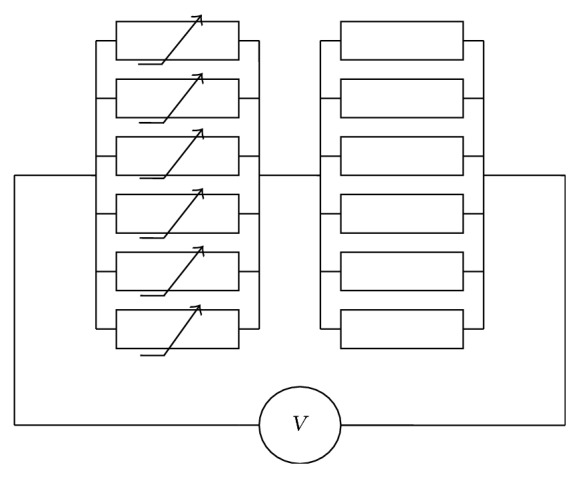
Electrical scheme of the GJ channel composed of two hemichannels each formed of 6 connexins/subgates. *V*_*j*_ influence both hemichannels but only Cxs on the left operate between open, closed, and deep closed states, while Cxs on the right are always open.

We assume that in this GJ model only subgates in the left hemichannel operate [4], while subgates in the right hemichannel are always open (see Figure 3).

**Figure 4:**
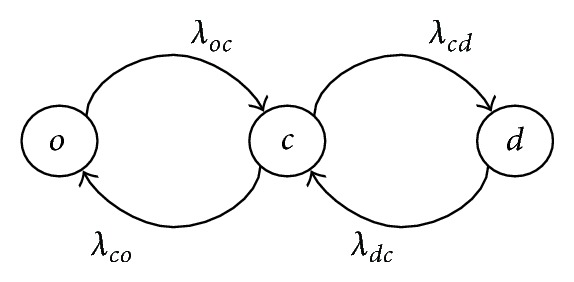
Three-state transition graph of a subgate.

We also assume that each subgate operates between open (*o*), closed (*c*), and deep-closed (*d*) states. The transition among these states is presented in Figure 4.

In this case our SAN model consists of a single automaton *A*_3_^(*l*)^ (subscript value 3 denotes that each subgate has 3 possible states), which describes the left hemichannel. If we assume that states of the left hemichannel are numbers of open and closed gates, denoted by *n*_*o*_ and *n*_*c*_, respectively, then the state-space of a system consists of 2-tuples (*n*_*o*_, *n*_*c*_) satisfying the following inequality:

(31) $$\begin{array}{c} n_{o}+n_{c}\leq 6. \end{array}$$

It follows from (31) that the state-space has the size of 28. From a state (*n*_*o*_, *n*_*c*_) automaton can go to the state (*n*_*o*_ + 1, *n*_*c*_ − 1) with transition rate *n*_*c*_ · *λ*_*co*_; to the state (*n*_*o*_ − 1, *n*_*c*_ + 1) with transition rate *n*_*o*_ · *λ*_*oc*_; to the state (*n*_*o*_, *n*_*c*_ + 1) with transition rate (6 − *n*_*o*_ − *n*_*c*_) · *λ*_*dc*_; and finally to the state (*n*_*o*_, *n*_*c*_ − 1) with transition rate *n*_*c*_ · *λ*_*cd*_.

An infinitesimal generator **Q**_(3)_^(*l*)^ of the left hemichannel (as well as the global system generator, i.e., **Q**_(3)_^(*l*)^ = **Q**_(3)_^(6)^) has the same block tridiagonal structure as in (14):

(32) $$\begin{array}{c} Q_{\left(3\right)}^{\left(6\right)}=\left(\begin{array}{ccccccc} Q_{11} & Q_{12} & 0 & 0 & 0 & 0 & 0\\ Q_{21} & Q_{22} & Q_{23} & 0 & 0 & 0 & 0\\ 0 & Q_{32} & Q_{33} & Q_{34} & 0 & 0 & 0\\ 0 & 0 & Q_{43} & Q_{44} & Q_{45} & 0 & 0\\ 0 & 0 & 0 & Q_{54} & Q_{55} & Q_{56} & 0\\ 0 & 0 & 0 & 0 & Q_{65} & Q_{66} & Q_{76}\\ 0 & 0 & 0 & 0 & 0 & Q_{76} & Q_{77} \end{array}\right). \end{array}$$

However, all the blocks in (32) including zero-entry blocks are of varying sizes. In general, block entry *ij* of the infinitesimal generator is of size (8 − *i*) × (8 − *j*). For example, diagonal blocks **Q**_*ii*_ are square matrices of size (8 − *i*) each and have the following tridiagonal form:

(33) $$\begin{array}{c} Q_{ii}=\left(\begin{array}{ccccc} {}* & \left(7-i\right)\lambda_{dc} & \vdots & 0 & 0\\ \lambda_{cd} & * & \vdots & 0 & 0\\ 0 & 2\lambda_{cd} & \vdots & 0 & 0\\ \cdots & \cdots & \ddots & \cdots & \cdots \\ 0 & 0 & \vdots & 2\lambda_{dc} & 0\\ 0 & 0 & \vdots & * & \lambda_{dc}\\ 0 & 0 & \vdots & \left(7-i\right)\lambda_{cd} & * \end{array}\right), \end{array}$$

where *λ*_*dc*_ = *λ*_*dc*_(*V*_left_(*n*_*o*_, *n*_*c*_)) = *λ*_*dc*_(*V*_left_(*i*, *j*)) and *λ*_*cd*_ = *λ*_*cd*_(*V*_left_(*n*_*o*_, *n*_*c*_)) = *λ*_*cd*_(*V*_left_(*i*, *j*)) and *j* is the row in a block **Q**_*ii*_ where *λ*_*dc*_ or *λ*_*cd*_ appears.

All upper diagonal blocks **Q**_*i*,*i*+1_ have the following structure (zero-entries row vector augmented with diagonal matrix):

(34) $$\begin{array}{c} Q_{i,i+1}=\left(\begin{array}{cccc} 0 & 0 & \vdots & 0\\ \lambda_{co} & 0 & \vdots & 0\\ 0 & 2\lambda_{co} & \cdots & 0\\ \vdots & \vdots & \vdots & 0\\ 0 & 0 & \vdots & \left(7-i\right)\lambda_{co} \end{array}\right), \end{array}$$

where *λ*_*co*_ = *λ*_*co*_(*V*_left_(*n*_*o*_, *n*_*c*_)) = *λ*_*co*_(*V*_left_(*i* − 1, *j* − 1)) and *j* is the row in a block **Q**_*i*,*i*+1_ where *λ*_*co*_ appears.

Similarly, lower diagonal blocks **Q**_*i*+1,*i*_ are diagonal matrices augmented with a zero-entry column and may be written as follows:

(35) $$\begin{array}{c} Q_{i+1,i}=\left(\begin{array}{ccccc} 0 & \left(i-1\right)\lambda_{oc} & 0 & \vdots & 0\\ 0 & 0 & \left(i-1\right)\lambda_{oc} & \vdots & 0\\ \cdots & \cdots & \cdots & \ddots & \cdots \\ 0 & 0 & 0 & \vdots & \left(i-1\right)\lambda_{oc} \end{array}\right), \end{array}$$

where *λ*_*oc*_ = *λ*_*oc*_(*V*_left_(*n*_*o*_, *n*_*c*_)) = *λ*_*oc*_(*V*_left_(*i* − 1, *j* − 1)) and *j* is the row in a block **Q**_*i*+1,*i*_, where *λ*_*co*_ appears.

The first problem arising in steady-state calculation is that the blocks are of different sizes. It means that the recursive procedure, which was most efficient for GJ models with 12 two-state Cxs, cannot be applied in this case. This leaves only banded Gaussian elimination and block Gauss-Seidel methods for further detailed consideration.

#### 3.4.1. Banded Gaussian Elimination for Steady-State Solution of CTMC Model of GJ Channel Containing 6 Three-State Subgates

It is easy to see from (32) that **Q**_(3)_^(6)^ has the same bandwidth *m* = 15 as **Q**_(2)_^(12)^ but the size of state-space *n* = 28 is different, in this case. So, according to the complexity evaluation presented in [24], the steady-state solution by using banded Gaussian elimination requires only about 4-fold less CPU time than standard Gaussian elimination for dense linear system. Thus, the difference is not as pronounced as in the previous example.

#### 3.4.2. Block Gauss-Seidel Elimination for Steady-State Solution of CTMC Model of GJ Channel Containing 6 Three-State Gates

The application of block Gauss-Seidel is very similar to that for GJ model with 12 two-state Cxs. Basically, the same iterative procedure (28) can be applied; however blocks are of different sizes in this case. Thus, even though the same basic operations with the same complexity as presented in Table 2 are used for this model, one must evaluate varying sizes of blocks in the matrix **Q**_(3)_^(6)^. For example, instead of solving 7 tridiagonal systems, each of size 7, one needs only to solve one system of size 7, one of size 6, and so forth.

This gives an estimated complexity of 196 per single outer iteration with the block Gauss-Seidel method. Thus, block Gauss-Seidel outperforms standard Gaussian elimination if less than 80 outer iterations are needed. It also outperforms banded Gaussian elimination if less than 18 outer iterations are needed.

As for the 12 two-state Cxs GJ model, we evaluated the number of outer iterations necessary to achieve required precision. The convergence speed is presented in Table 5.

Thus, it is less feasible to use block Gauss-Seidel method than banded Gaussian elimination if higher than 10^−5^ precision is used, though it is more efficient than standard Gaussian elimination even with 10^−15^ precision.

However, the efficiency of block Gauss-Seidel becomes higher if the repetitive model solution is performed. A similar experiment as with the 12 two-state Cxs model was performed for 6 three-state subgates GJ model. The results are presented in Table 6.

The results showed that block Gauss-Seidel becomes more efficient than banded Gaussian elimination under reasonable 10^−6^ precision if repetitive calculations are performed.

### 3.5. CTMC Model of the GJ Channel Containing 12 Three-State Subgates

Here, we consider that in the model each subgate of both hemichannels operates between open (*o*), closed (*c*), and deep closed (*d*) states. Thus, its electrical scheme is the same as presented in Figure 1, while the state graph of the subgate is as presented in Figure 3.

We model this type of GJ by SAN, containing two automata. We assume that *A*_3_^(*l*)^ describes the left hemichannel, while *A*_3_^(*r*)^ describes the right one (again, subscript values 3 denote the fact that each subgate has 3 possible states). The states of both automata *A*_3_^(*l*)^ and *A*_3_^(*r*)^ are 2-tuples (*n*_*o*_^(*l*)^, *n*_*c*_^(*l*)^) and (*n*_*o*_^(*r*)^, *n*_*c*_^(*r*)^), respectively. Here *n*_*o*_^(*l*)^ and *n*_*c*_^(*l*)^ denote the number of open and closed subgates on the left hemichannel, while *n*_*o*_^(*r*)^ and *n*_*c*_^(*r*)^ denote the number of closed and open gates on the right one. They also must satisfy the following inequalities:

(36) $$\begin{array}{c} n_{o}^{\left(l\right)}+n_{c}^{\left(l\right)}\leq 6,\;\;n_{o}^{\left(r\right)}+n_{c}^{\left(r\right)}\leq 6. \end{array}$$

Transitions among states in each hemichannel are analogous to those of the previous model. Similarly, infinitesimal generators **Q**_3_^(*l*)^ and **Q**_3_^(*r*)^ can be written as in (32). Thus global infinitesimal generator **Q**_3_^(12)^ may be expressed as

(37) $$\begin{array}{c} Q_{\left(3\right)}^{\left(12\right)}=Q_{\left(3\right)}^{\left(l\right)}\underset{g}{⊗}I_{28}+I_{28}\underset{g}{⊗}Q_{\left(3\right)}^{\left(r\right)}. \end{array}$$

**Figure 5:**
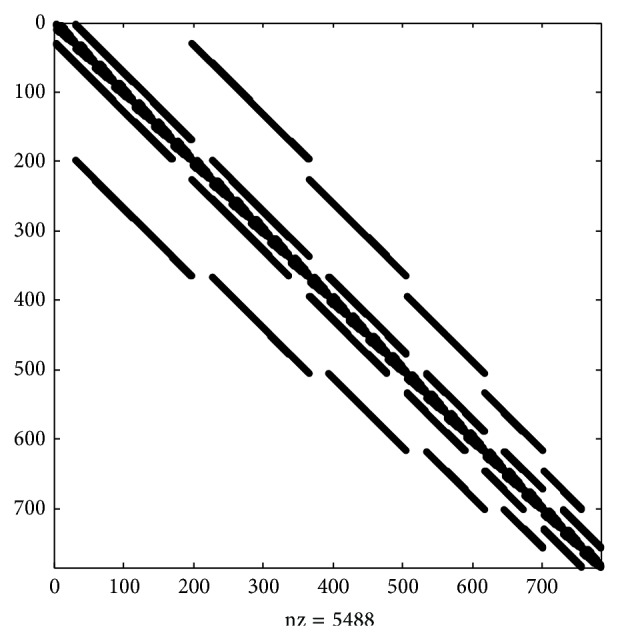
Nonzero entry structure of global system generator **Q**_(3)_^(12)^ of GJ channel composed of two hemichannels formed of 6 connexins with 3 states.

It is easy to see from (37) that **Q**_(3)_^(12)^ has the size of 748; thus it is a much larger model than in previous examples. It is not possible due to the size of the matrix to present its full block structure. However, its nonzero entry structure, which was obtained by using* spy*() function from MATLAB package, is presented in Figure 5.

It is also possible to analyze its structure based on the Kronecker representation of system descriptor (37). As can be seen from Figure 5, it consists of 5 layers of blocks, all of which are square matrices of size 28. Diagonal blocks **Q**_*ii*_ are all block tridiagonal matrices, which may be written as follows:

(38) $$\begin{array}{c} Q_{ii}=q_{ii}⊗I_{7}+Q_{\left(3\right)}^{\left(6\right)}. \end{array}$$

Here *q*_*ii*_ denotes the *i*th diagonal entry of the matrix **Q**_(3)_^(6)^.

It follows from (38) that **Q**_*ii*_ has the same nonzero entries structure as **Q**_(3)_^(6)^. Matrix **Q**_(3)_^(12)^ also has two layers of blocks in lower and upper parts. For simplicity, we call them inside-lower (inside-upper) and outside-lower (outside-upper) layers. All of these blocks are diagonal matrices and can be written as

(39) $$\begin{array}{c} Q_{ij}=q_{ij}⊗I_{28}, \end{array}$$

where *q*_*ij*_ denotes the *ij*th entry of matrix **Q**_(3)_^(6)^.

Since matrix **Q**_(3)_^(12)^ is not tridiagonal, it leaves banded Gaussian elimination and block Gauss-Seidel methods for more detailed consideration.

#### 3.5.1. Banded Gaussian Elimination for Steady-State Solution of CTMC Model of GJ Channels Containing 12 Three-State Subgates

Even though matrix (38) is banded, its bandwidth 393 is much bigger in both absolute and relative (*m* exceeds half of the size of *n*) values than that of 12 two-state Cxs GJ model. It requires about 5 times less CPU time than standard Gaussian elimination to calculate steady-state probabilities.

#### 3.5.2. Block Gauss-Seidel Algorithm for Steady-State Solution of CTMC Model of GJ Channels Containing 12 Three-State Subgates

Analyzing the zero-entries structure of (38) allows for adaptation of the block Gauss-Seidel algorithm for **Q**_(3)_^(12)^ model. Basically, in one outer iteration step one needs to solve 28 inner iterations—each of them is a linear system of size 28:

(40) $$\begin{array}{c} \pi_{i}^{\left(k+1\right)}=bQ_{ii}^{-1},\;i=\bar{1,28}, \end{array}$$

where row vector **b** might consist of up to four vectors of form ***π***_*r*_^(*k* + 1)^ **Q**_*l*,*m*_ or ***π***_*r*_^(*k*)^ **Q**_*l*,*m*_. In general, the number of arithmetic operations necessary to perform one outer iteration of the block Gauss-Seidel algorithm for **Q**_(3)_^(12)^model is presented in Table 7 (size *n* in this case is equal to 28).

Thus, the block Gauss-Seidel algorithm becomes more efficient than standard Gaussian elimination if it requires less than 5954 outer iterations. Actual numbers of outer iterations necessary to achieve the required precision at 40 mV transjunctional voltage level are presented in Table 8.

Basically the convergence speed of block Gauss-Seidel for solving steady-state probabilities of the 12 three-state subgates GJ model is comparable to that of previous models. Also, block Gauss-Seidel is much more efficient than even banded Gaussian elimination with any precision. As in previous cases, it outperformed other standard iterative and projection methods.

For example, it required less than 40 milliseconds of CPU time to calculate steady-state probabilities with 10^−6^ precision with MATLAB while its implementation in C++ required about 18 milliseconds.

The repetitive model solution showed the same effect of iterative algorithms on convergence speed as in previous examples. The results are presented in Table 9.

Overall, about 40 percent less CPU time was required to estimate steady-state probabilities in the whole range of transjunctional voltage by using, if previous solutions were used as the first iteration vector.

## 4. Conclusion

SAN formalism is efficient for the creation of models of GJ voltage gating, because system description is relatively simple, and the building and storing of the infinitesimal generator is very rapid. We used SAN to create equivalent CTMC model due to the complexity of estimation of functional transition rates. Unlike a PLA approach, which we have used earlier for CTMC modelling of GJs [8], SAN and Kronecker algebra operations help to get more insight into the structure of infinitesimal generator matrix.

Infinitesimal generators of GJ models have a distinct block structure that allows selecting most efficient numerical methods. For example, application of banded Gaussian method lowers CPU time at least 4-fold as compared to standard Gaussian elimination, which typically is applied for estimation of steady-state probabilities of DTMC models with dense matrices.

Iterative methods are very suitable for GO of gating parameters, since it requires numerous simulations at different *V*_*j*_s. In particular, the block Gauss-Seidel method can be applied very successfully, since it also benefits from the block structure of the infinitesimal generator. The implementations proposed in this study outperformed even direct methods used in calculation of steady-state probabilities.

We assume that implementation of different numerical methods, for example, using the most advanced numerical techniques in SAN modelling, could lead to even better results. This could help to reduce computational time in search of most adequate mathematical models in describing voltage gating of GJs.

## Figures and Tables

**Pseudocode 1:**
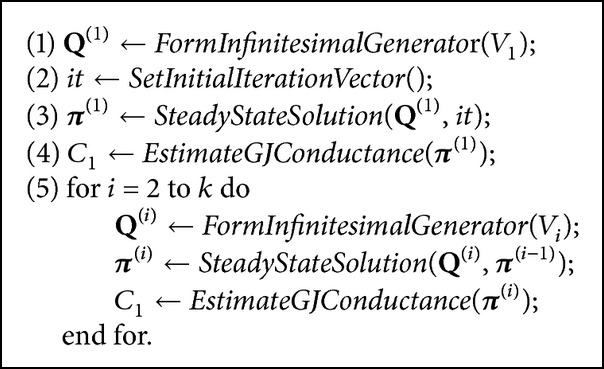


**Table 1:** Evaluation of the number of arithmetic operations necessary to implement the recursive procedure for GJ model with 12 two-state subgates.

Part of an algorithm	Number of times	Number of arithmetic operations
Matrix + matrix	11	*n* ^2^
Matrix ∗ matrix	6	*n* ^3^ − *n* ^2^
Matrix ∗ diagonal matrix	13	*n* ^2^
Vector ∗ diagonal matrix	6	*n*
Vector ∗ tridiagonal matrix	7	5*n*
Solve dense system	1	(4*n* ^3^ + 9*n* ^3^ − 13*n* ^3^)/6

**Table 2:** Number of arithmetic operations necessary to implement a single outer iteration of block Gauss-Seidel algorithm for GJ model with 12 two-state subgates.

Part of an algorithm	Number of times	Number of arithmetic operations
Vector + vector	5	*n *
Vector ∗ tridiagonal matrix	12	5*n *
Solve tridiagonal system	7	8*n *
Check for convergence	1	*n*

**Table 3:** Number of outer iterations necessary to achieve the required precision by using block Gauss-Seidel algorithm for GJ model with 12 two-state subgates.

Precision	Number of iterations
10^−4^	15
10^−5^	18
10^−6^	20
10^−7^	23
10^−8^	25
10^−9^	28
10^−10^	31
10^−11^	33
10^−12^	36
10^−13^	39
10^−14^	41
10^−15^	44

**Table 4:** Number of outer iterations for repetitive model solution by using block Gauss-Seidel algorithm for GJ model with 12 two-state subgates.

Voltage, mV	Number of iterations	
	Method I	Method II
20	15	6
40	20	10
60	26	16
80	19	11
100	11	5

**Table 5:** Number of outer iterations necessary to achieve the required precision by using block Gauss-Seidel algorithm for GJ model with 6 three-state subgates.

Precision	Number of iterations
10^−4^	14
10^−5^	20
10^−6^	25
10^−7^	30
10^−8^	35
10^−9^	40
10^−10^	45
10^−11^	50
10^−12^	56
10^−13^	61
10^−14^	66
10^−15^	71

**Table 6:** Number of outer iterations for repetitive model solution by using block Gauss-Seidel algorithm for GJ model with 6 three-state subgates.

Voltage, mV	Number of iterations	
	Method I	Method II
20	12	7
40	18	15
60	20	9
80	14	4
100	12	3

**Table 7:** Number of operations necessary to perform a single outer iteration of block Gauss-Seidel algorithm for GJ model with 12 three-state subgates.

Part of an algorithm	Number of times	Number of arithmetic operations
Vector + vector	56	*n*
Vector ∗ diagonal matrix	84	*n*
Solve tridiagonal system	28	8*n*
Check for convergence	1	*n*

**Table 8:** Number of outer iterations necessary to achieve the required precision by using block Gauss-Seidel algorithm for GJ model with 12 three-state subgates.

Precision	Number of iterations
10^−4^	14
10^−5^	23
10^−6^	32
10^−7^	40
10^−8^	49
10^−9^	58
10^−10^	66
10^−11^	75
10^−12^	83
10^−13^	92
10^−14^	101
10^−15^	109

**Table 9:** Number of outer iterations for repetitive model solution by using block Gauss-Seidel algorithm for GJ model with 6 three-state subgates.

Voltage, mV	Number of iterations	
	Method I	Method II
20	24	15
40	32	25
60	30	23
80	18	8
100	12	3
